# A Thermal Analytical Study of LEGO^®^ Bricks for Investigating Light-Stability of ABS

**DOI:** 10.3390/polym15153267

**Published:** 2023-07-31

**Authors:** Francesca Sabatini, Silvia Pizzimenti, Irene Bargagli, Ilaria Degano, Celia Duce, Laura Cartechini, Francesca Modugno, Francesca Rosi

**Affiliations:** 1 Institute of Chemical Science and Technologies “G. Natta” (CNR-SCITEC), Via Elce di Sotto 8, 01628 Perugia, Italy; f.sabatini4@gmail.com (F.S.); irene.bargagli@studenti.unipg.it (I.B.); laura.cartechini@cnr.it (L.C.); francesca.rosi@cnr.it (F.R.); 2 Department of Chemistry and Industrial Chemistry, University of Pisa, Via G. Moruzzi 13, 56124 Pisa, Italy; silvia.pizzimenti@gmail.com (S.P.); celia.duce@unipi.it (C.D.)

**Keywords:** ABS, TGA, DSC, EGA-MS, Py-GC/MS, degradation

## Abstract

Acrylonitrile butadiene styrene (ABS) is a thermoplastic polymer widely used in several everyday life applications; moreover, it is also one of the most employed plastics in contemporary artworks and design objects. In this study, the chemical and thermal properties of an ABS-based polymer and its photo-degradation process were investigated through a multi-analytical approach based on thermal, mass spectrometric and spectroscopic techniques. LEGO^®^ building blocks were selected for studying the ABS properties. First, the composition of unaged LEGO^®^ bricks was determined in terms of polymer composition and thermal stability; then, the bricks were subjected to UV–Vis photo-oxidative-accelerated ageing for evaluation of possible degradation processes. The modifications of the chemical and thermal properties were monitored in time by a multi-technique approach aimed at improving the current knowledge of ABS photodegradation, employing pyrolysis online with gas chromatography and evolved gas analysis, coupled with mass spectrometric detection (Py-GC-MS and EGA-MS), differential scanning calorimetry (DSC), thermogravimetric analysis (TGA), and corroborated by external reflection FT-IR spectroscopy. The multimodal approach provided new evidence on the two-step degradation pathway proposed for ABS, defining molecular markers for polybutadiene oxidation and styrene-acrylonitrile depolymerization. Moreover, the results highlighted the feasibility of correlating accurate compositional and thermal data acquired by bulk techniques with external reflection FT-IR spectroscopy as a non-invasive portable tool to monitor the state of conservation of plastic museum objects in-situ.

## 1. Introduction

The rapid and drastic growth in global plastic production in the 20th century and the continuous implementation of new synthetic polymer formulations have made plastic one of the most versatile materials, employed in an enormous variety of application areas. Artistic production and heritage collections were not unaffected by the introduction of synthetic plastic materials in the wider market; however, starting from the 1930s, an increasing number of objects made of synthetic polymers, such as artworks, design objects, furniture and toys were produced, that are, today, conserved in museums and collections dedicated to contemporary art, design, science, and fashion. Paints, coatings, adhesives and consolidants consisting of synthetic polymers are also encountered in collections as restoration materials, and often used for conservation [1,2,3,4,5].

Amongst the many types of plastics, polyurethane, unsaturated polyester, plasticized poly(vinyl chloride), poly(methyl methacrylate) and acrylonitrile butadiene styrene (ABS) are those used most frequently in artworks, artistic installations and design objects [6]. Non-invasive analytical investigations carried out in the last few years at *Triennale Milano Museo del Design Italiano* [7] and *Museo Kartell* within the MOLAB access activity of the European Research Infrastructure for Heritage Science (ERIHS) [8], revealed that ABS is one of the most employed materials in a wide variety of design objects.

ABS is a thermoplastic polymer constituted by three monomeric units: acrylonitrile (AN), butadiene (BU), and styrene (S) (Figure S1, Supplementary Materials). Acrylonitrile imparts heat and chemical resistance, and surface hardness to the system, butadiene confers plasticity and impact resistance, while styrene provides processibility, rigidity, and strength [9]. One of the most common methods to manufacture ABS is grafting a styrene-acrylonitrile (SAN) copolymer onto polybutadiene (PB), resulting in long chains of PB grafted with side-chains of SAN [10] (Figure S1, Supplementary Materials). Thus, ABS can be considered a heterogeneous plastic material constituted by a dispersed rubbery phase (PB) and a glassy matrix copolymer (SAN) [11].

Thermoanalytical techniques have been widely employed for investigating the thermal and photo stability of ABS, the thermal-degradation kinetics, and the mechanical and chemical properties. In particular, thermogravimetric analysis (TGA) was employed to test the effects of different experimental conditions (i.e., heating rates, gas conditions) on commercial types of ABS to evaluate the thermal decomposition steps and calculate the relative activation energy [12,13,14,15,16,17,18]. Thermogravimetric analysis was also coupled with Fourier transform infrared (TGA/FTIR) [18,19,20,21,22] or mass spectrometry (TGA/MS) [21,23] to investigate the products evolved in the heating process. Differential scanning calorimetry (DSC) enabled the study of the thermal behavior of ABS [13,24,25], evaluating the role played by the presence of antioxidants [26,27] and outdoor and indoor exposure for prolonged periods of time [11,27]. Nevertheless, the application of such techniques cannot provide a detailed molecular knowledge of the material composition and the use of pyrolysis coupled with gas chromatography and mass spectrometry (Py-GC/MS) was needed for this aim. Py-GC/MS has been successfully applied for the chemical characterization of polymers and additives [28,29,30,31,32,33,34,35], and although several studies have been devoted to the analysis of ABS, they are mainly focused on the possible release of toxic compounds [10,36] or on the application of co-pyrolysis on blends of ABS with other plastics for the investigation of the mutual interactions and the properties of the single material in the resulting blends [18,37,38]. Double shot pyrolysis coupled with gas chromatography and mass spectrometry (DS-Py-GC/MS) [23] and evolved gas analysis coupled with mass spectrometry (EGA-MS) [12] have rarely been applied to ABS and no studies have been dedicated to the combined evaluation of the variation in the thermal–mechanical properties or in the composition of artificially photoaged ABS.

In the present work, ABS was used as plastic material for a pilot study aimed at the definition of a novel, integrated and comprehensive protocol based on state-of-the-art thermal analytical techniques for the chemical characterization of plastic materials and the determination of their photo-stability. LEGO^®^ bricks, cheap and widely available toys made of ABS, were selected for optimizing a combined analytical approach based on TGA, EGA-MS, DSC and Py-GC-MS to study the thermal behavior and compositional changes in the ABS during photo-accelerated ageing. Despite the available literature on ABS from previous thermoanalytical studies, the approach used provided new evidence of the occurring degradation process and allowed us to define oxidation markers. The results were corroborated with the data collected by external reflection FT-IR spectroscopy to monitor compositional variations occurring at the sample surface.

From a wider perspective, the correlation between the data obtained by non-invasive techniques, deployable in-situ, and micro-destructive analysis will enable the application of a tailored protocol to monitor plastic materials in museums and collections (e.g., design objects, but also conservation and consolidation materials). The correlation between detailed and complementary information will enable us to indirectly obtain information about the thermal and possibly mechanical properties of the materials, which is crucial for the provision of reliable and effective information about the conservation state of the artworks.

## 2. Materials and Methods

### 2.1. Samples

A set of contemporary LEGO^®^ bricks (The Lego Group) was purchased from a local store in Italy, in July 2022 (batch number 11019). Light green and white bricks were selected for this study. Powdered white and green samples (labeled in the following as Wt0, Gt0), obtained by manual scraping, were analyzed with EGA-MS, DS-Py-GC-MS, TGA and DSC. Both green and white samples contain titanium dioxide (TiO_2_) as pigment and/or filler (Raman spectra are reported in Figure S2, Supplementary Materials), and the green brick also contained phthalocyanine-based pigments (Pigment Green 7—PG7 and Pigment Green 36—PG36) (Raman spectra are reported in Figure S2, Supplementary Materials). The investigation of the ABS-photo ageing was carried out on the green brick samples only, while the white ones were analyzed as control, showing the same profile (Figure S3, Supplementary Materials).

Intact LEGO^®^ bricks and powdered green samples were artificially aged using a dedicated optical bench consisting of a Cermax filtered Xenon lamp (300 W, working λ > 320 nm), placing the bricks and the powders in a sample holder on the bench, 50 cm from the light source. At such a distance, the irradiation image was homogenous within about 10% variation range. The measured temperature during the irradiation was 30 °C. The samples were artificially aged during a wide time span (from 8 to 1390 h) and analyzed with the above mentioned techniques to characterize compositional and thermal property variations at the early and prolonged stages of light ageing, with the following monitoring steps: 0 h (Gt0), 8 h (Gt8h), 24 h (Gt24h), 36 h (Gt36h), 48 h (Gt48h), 120 h (Gt120h), 240 h (Gt240h) and 1390 h (Gt1390h). Spectroradiometric measurements performed on the sample holder allowed us to determine the total irradiance (Փ_0_ = 60 W/m^2^) and illuminance (Em_0_ = 40,980 lux) after 1390 h for each irradiated sample. Moreover, external reflection FT-IR spectroscopy was performed on the irradiated side of the intact bricks (spectra and working conditions in Figure S4, Supplementary Materials).

### 2.2. Apparatus

#### 2.2.1. Differential Scanning Calorimetry

DSC experiments were carried out by a Discovery DSC, model 250 (TA Instruments; New Castle, DE, USA) under nitrogen gas flow 50 mL/min. The DSC was calibrated with indium. For each sample, 1.0–1.8 mg was weighed and hermetically sealed in aluminum DSC pans. Two methods were used, on each sample. In the first method, temperature was scanned by a double heating–cooling cycle from 20 to 200 °C with a heating rate of 20 °C/min and a cooling rate of 30 °C/min. The glass transition temperature (midpoint type: half height) was evaluated from the second heating scan. In the second method, the temperature was scanned by a double cooling–heating cycle from 20 to −80 °C with a cooling rate of 5 °C/min and a heating rate of 20 °C/min. An empty pan was used as a reference. TRIOS software (version 5.2) was used to carry out the data analysis of the thermograms.

#### 2.2.2. Thermogravimetric Analysis

A thermobalance, model Q5000IR (TA Instruments; New Castle, DE, USA) was used for TGA investigations. Temperature calibration was based on the Curie point of paramagnetic metals. A multipoint calibration with five Curie points from reference materials (Alumel, Ni, Ni 83%-Co 17%, Ni 63%-Co 37%, Ni 37%-Co 63%) was performed. TGA measurements were carried out at a heating rate of 10 °C/min from 25 to 700 °C under nitrogen flow (25 mL/min). The amount of sample in each measurement varied between 0.8 and 1.2 mg. TA Universal Analysis software was used in the data analysis of the thermograms. Triplicate analyses provided a 0.1% relative standard deviation.

#### 2.2.3. Evolved Gas Analysis Coupled with Mass Spectrometry

Experiments were performed with an EGA/PY-3030D microfurnace pyrolyzer (Frontier Laboratories Ltd., Japan) coupled to a 6890 gas chromatograph and a 5973 mass spectrometric detector (Agilent Technologies, Santa Clara, CA, USA). A deactivated stainless-steel capillary tube (UADTM-2.5 N, 0.15 mm in diameter × 2.5 m length, Frontier Laboratories Ltd., Fukushima, JP) was used to connect the injection port to the mass spectrometer. The products evolved from the samples over the temperature range were transferred to the mass spectrometer, ionized, and analyzed as a function of time.

About 0.2 mg of each sample was placed in a clean stainless-steel cup and inserted into the microfurnace. The temperature program set for the microfurnace was: initial temperature 50 °C then 10 °C/min up to 800 °C. The interface temperature was kept 100 °C higher than the furnace temperature up to 300 °C. The injection port operated at 280 °C, with a 1:20 split ratio. The chromatographic oven was kept at 300 °C during the whole EGA analysis. The analyses were performed in constant flow mode at 1.0 mL/min (He, purity 99.995%). The deactivated stainless-steel capillary tube was kept at 300 °C. The mass spectrometer was operated in EI positive mode (70 eV, *m*/*z* range 35–600). The ion source and quadrupole temperatures were 230 and 150 °C, respectively. The data collected were processed by Agilent MSD ChemStation (version D.02.00.275).

The variability of the peak temperature (Tp), defined as the temperature corresponding to the maxima of the total ion thermogram (TIT) profile, was evaluated for all the inter-day replicates of Gt120 h by *t*-test. The triplicate experiments did not show any variation in the Tp value.

#### 2.2.4. Double Shot Pyrolysis Coupled with Gas Chromatography and Mass Spectrometry

Analyses were performed using an EGA/PY-3030D micro-furnace pyrolyzer coupled to an 8890 gas chromatograph and a 5977 mass selective detector (Agilent Technolog, Palo Alto, CA, USA). About 150 µg of each sample was placed into a clean stainless-steel cup and inserted into the preheated microfurnace. The pyrolysis temperatures for the double shot experiment were selected on the basis of the thermal regions highlighted in the EGA profile of the specific investigated material. Thus, it was possible to characterize the products evolved or pyrolyzed at each one of two thermal regions separately, enhancing the discrimination capability of the technique and simplifying the comparison of the obtained molecular profiles with the literature [28]. To obtain information on the first region, the temperature of the first Py-GC-MS shot was set at 300 °C, while for the second Py-GC-MS shot, the temperature was set at 600 °C.

The pyrolyzer interface and the GC injector were operated at 280 °C, with a 10:1 split ratio. Separation of the pyrolysis products was achieved with an HP-5MS fused silica capillary column (95% dimethyl, 5% phenyl polysiloxane, 30 m × 0.25 mm, film thickness 0.25 µm, Agilent Technologies, Palo Alto, CA, USA) and helium as carrier gas (1 mL/min). The chromatographic program was: initial temperature 40 °C for 6 min, 20 °C/min to 310 °C for 40 min. The mass spectrometer was operated in EI positive mode (70 eV, *m*/*z* range 35–600). The ion source was kept at 230 °C, while the quadrupole analyzer at 150 °C. The data collected were interpreted with the aid of the NIST Mass Spectral Search Program (version 2.0), mass spectral libraries and F-Search MP software (version 3.6.0). Data acquisition and interpretation were performed with MassHunter (version 10.0).

## 3. Results and Discussion

### 3.1. Differential Scanning Calorimetry

**Figure 1:**
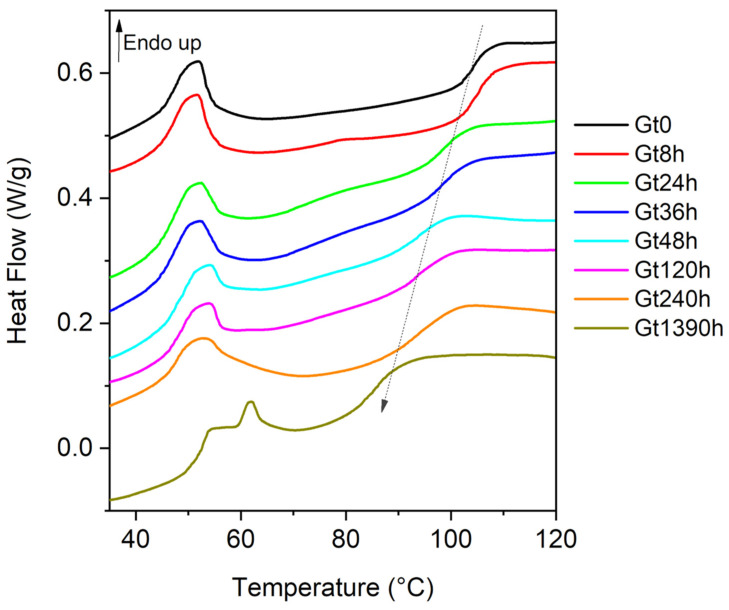
Experimental DSC curves of the heat flow normalized in the second heating scan vs. temperature for green samples at different ageing times (Gt0, Gt8h, Gt24h, Gt36h, Gt48h, Gt120h, Gt240h and Gt1390h). The Tg values are reported in Table 1. Upward peaks are endothermic phenomena (↑ Endo up). All curves are presented in the same scale and are stacked for purposes of clarity.

The DSC profiles of the unaged white and green bricks (Wt0, Gt0) in the 30–120 °C range were different (Figure S5, Supplementary Materials). The curve acquired for Wt0 was characterized by a first glass transition with a very low intensity at 61 °C and a second one at 100 °C (Figure S5, Supplementary Materials). The Gt0 curve showed a glass transition at 104 °C, due to the SAN [13,24,39] (Figure 1) and one reversible endothermic peak (↑ Endo up) in the temperature range 45–60 °C, not unequivocally ascribable to a specific component of the sample (possibly hindering the first glass transition shown in Wt0). Analyses performed on the phthalocyanines (PG7 and PG36) and TiO_2_, identified in green bricks, did not show such an endothermic peak, the cause of which at present remains unknown (see Figure S6, Supplementary Materials). DSC experiments were carried out for Wt0 and Gt0 samples also in the temperature range between −80 and 15 °C, but no signals below 0 °C and down to −80 °C were detected, indicating that the Tg of the PB phase, generally registered around −60/−80 °C [25,27], was not visible under the present experimental conditions.

In the aged samples, the Tg shifted towards lower temperatures (Figure 1 and Table 1), pointing at an increase in the plasticity of the material given by the chain scission in the SAN portion: the depolymerization leads to an increase in the mobility of the polymer chain resulting in a decrease in the Tg of the SAN portion. Since the Tg of the PB phase was not visible, the DSC curve provided information on the thermal behavior of the SAN portion of the ABS constituting the sample analyzed. The same alteration trend was reported after thermal ageing at 60 °C in the dark [40].

After 1390 h of photo-degradation treatment, a significant Tg decrease of about 19 degrees was observed, while a decrease of less than 2 degrees was reported in [40] after the same time (1400 h) of thermal ageing at 60 °C in the dark. The more extensive degradation of the polymer observed as a consequence of the photo-oxidation with respect to the thermal ageing described in [40] can be attributed to the more effective production of radicals during the irradiation of strongly UV-absorbing species, such as SAN, than by heating, and by the possible catalytic effect of copper-based pigments [40].

### 3.2. Thermogravimetric Analysis

**Figure 2:**
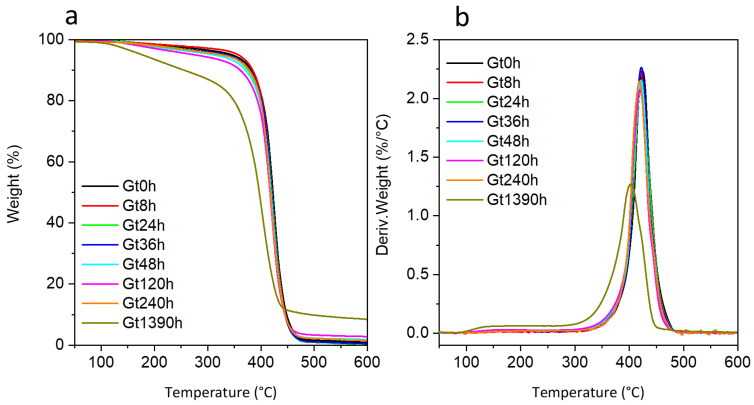
(**a**) Thermogravimetric (TG) curves and (**b**) their corresponding derivatives acquired under nitrogen flow at 10 °C/min heating rate for the green sample at different ageing times (Gt0, Gt8h, Gt24h, Gt36h, Gt48h, Gt120h, Gt240h and Gt1390h).

The TG curves collected for unaged white (Wt0) and green (Gt0) powdered bricks showed a single mass loss in the temperature range 350–500 °C (Figure 2 and Figure S3, Supplementary Material), in accordance with the literature [12,13,14,15,16,17,19]. This thermal degradation step, featuring the maximum temperature (Tmax) at 424 °C for Wt0 and at 426 °C for Gt0, is mostly due to the overlapping decompositions of the SAN and PB portions of the ABS polymer [12,15,16,41]. In the green sample, the decomposition of the two phthalocyanines (PG7 and PG36) is also included in this degradation step [42]. In contrast, titanium dioxide (TiO_2_), present in both green and white samples, is thermally stable in the investigated temperature range. The difference in the degradation step temperature for unaged white and green samples was not significative, suggesting the same polymer composition. It is known that phthalocyanines (among them PG7) can play a catalytic role in the cross-linking of the polybutadiene fraction of thermally aged ABS, resulting in embrittlement [40]. From the evidence collected in this work, the kind of pigment appears not to influence the thermal properties of the unaged system.

The thermal degradation of the SAN portion in ABS did not produce any residue at 600 °C [19,43]. Consistently, the small residue at 600 °C reported for Wt0 (1.0 wt%) and Gt0 (1.1 wt%) can be ascribed to the PB portion of ABS and to TiO_2_ [16]. Considering that for unpigmented ABS samples containing 40 wt% of PB, a residue of 9 wt% at 600 °C was reported [43], a PB content below 4 wt% can be roughly estimated in Wt0 and Gt0 (neglecting the TiO_2_ contribution to the residue). At longer irradiation times, an increase in the amount of residue was observed (Table 1), suggesting cross-linking phenomena in the sample. This is consistent with the thermo-oxidative degradation of the PB portion via radical oxidation, leading to a decrease in the double bond content and to the crosslinking of oxidized portions of the PB phase in ABS [40] that may account for a higher residue % after pyrolysis [40,44].

In the TG curves of the aged samples, the maximum temperature of the mass loss shifted towards lower temperatures with the increase in the irradiation time, indicating a decrease in thermal stability (Table 1). The same gradual decreasing trend and difference in temperature (ΔT) between Gt0 and Gt1390h was highlighted by EGA experiments carried out under the same experimental conditions in terms of inert atmosphere and heating rate (ΔT 21 °C for TGA and 18 °C for EGA-MS, see Table 1). A slight decrease could already be observed between 36 and 48 h. The FT-IR spectrum recorded in external reflection mode at 36 h (Figure S4, Supplementary Materials) showed the disappearance of the butadiene band at 965 cm^−1^ δ(C=C-H)_trans_ pointing at the loss of double bonds and the consequent start of photo-reactions, possibly correlating with the decrease in thermal stability observed in EGA-MS at this length of ageing time.

The gradual modification of the ν(C=O) at 1780–40 cm^−1^ with ageing is instead connected to polymer photo-oxidation [41], firstly occurring in the BU portion and successively, at the longest ageing time, in the SAN one. This is consistent with TGA data related to sample Gt1390 h in which the mass loss starts at about 100 °C confirming a decrease in thermal stability and suggesting the formation of low molecular weight compounds, and with the results of DSC analysis.

### 3.3. Evolved Gas Analysis Coupled with Mass Spectrometry

**Figure 3:**
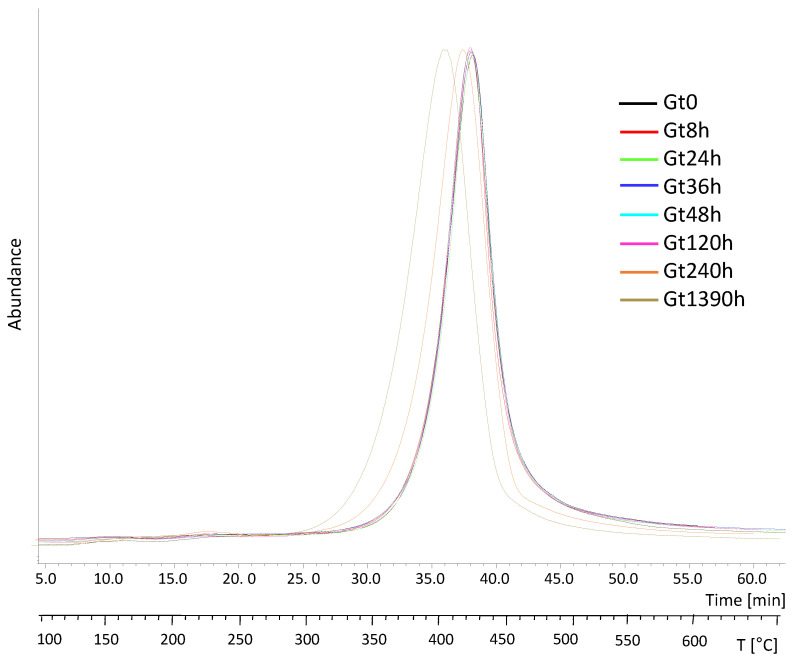
Total ion thermograms (TIT) of the samples from the green brick at different ageing times (Gt0, Gt8h, Gt24h, Gt36h, Gt48h, Gt120h, Gt240h and Gt1390h) normalized to 100.

The EGA profiles collected for the unaged white and green bricks (Wt0, Gt0) were both characterized by one main thermal region centered at 431 °C (peak temperature, Tp) and a very low intense band peaking at 230 °C (Figure 3). The EGA curves of all the aged green samples were nearly superimposable, registering a shift of the Tp at the longest ageing (ΔT 18 °C) (Table 1), pointing to a decrease in the thermal stability of the system, possibly due to depolymerization, consistent with the TGA results.

The average mass spectra of the two thermal regions visible in the total ion thermograms (TIT) of the Gt0 sample are reported in Figure S7a,b (Supplementary Materials). The spectra relative to the main thermal decomposition region (31.0–44.1 min, 360–491 °C, Figure S7b, Supplementary Materials) was dominated by ions ascribable to styrene [12] and to hybrid trimers of acrylonitrile-styrene (*m*/*z* = 39, 51, 78, 91, 104, 117, 144, 170). The thermal degradation region at lower temperatures (14.5–20.7 min, 195–257 °C, Figure S7a, Supplementary Materials) was characterized by ions (*m*/*z* = 44, 77, 91, 105, 129, 153) not straightforwardly assigned to specific compounds. Neither qualitative nor semiquantitative differences were evidenced in the average EGA mass spectra of the irradiated samples. Subsequently, to characterize each thermal region separately, double shot pyrolysis-GC/MS (DS-Py-GC/MS) was used, as described in Section 3.4.

### 3.4. Double Shot Pyrolysis Coupled with Gas Chromatography and Mass Spectrometry

**Figure 4:**
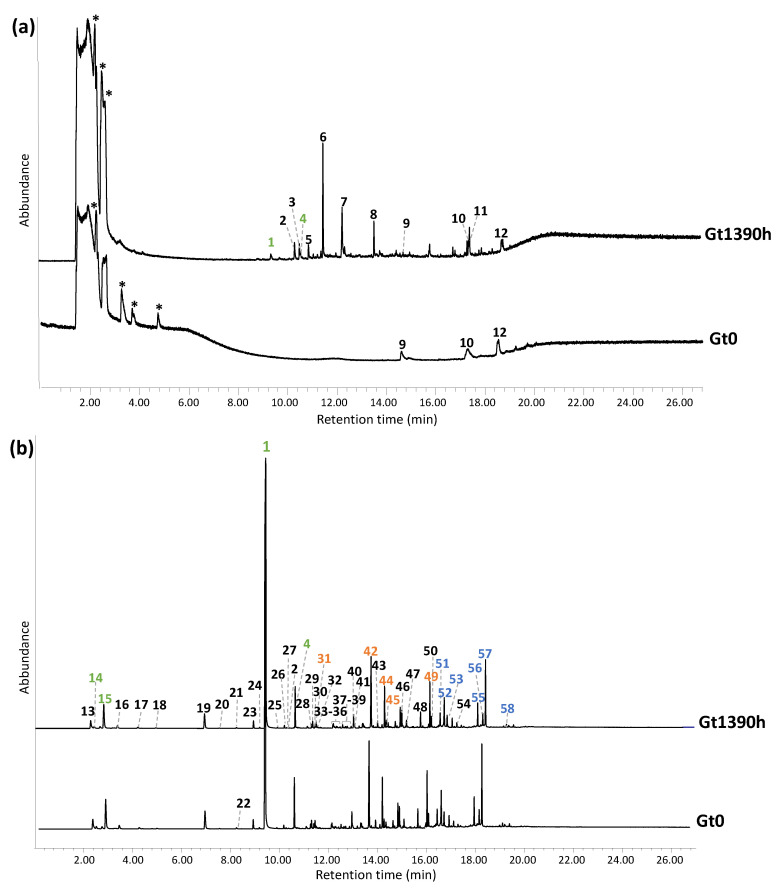
Py-GC/MS chromatograms obtained for (**a**) the first shot (300 °C) and (**b**) second shot (600 °C) of the unaged green sample (Gt0) and the 1390 h aged one (Gt1390h). The identification of the labelled peaks is reported in Table 2. The peaks specifically associated to the monomers are highlighted in green, while those related to dimers and trimers are in orange and blue, respectively. * = column impurities.

The temperatures of the two shots used for double shot Py-GC/MS were selected on the basis of the thermal features highlighted by the EGA-MS thermogram. Thus, for the first shot, a temperature of 300 °C was chosen to selectively desorb the most volatile compounds, such as additives or shorter polymer fragments, while the second shot was performed at 600 °C, to effectively pyrolyze the bulk polymer and ease comparison with the literature [28]. Neither qualitative nor semiquantitative differences were noticed between the pyrolysis profiles of Gt0 and Wt0, highlighting the same polymeric composition. This finding also helped us to rule out any possible interference in the pyrolysis process due to metal ion species contained in the phthalocyanine-based pigments. Conversely, significant qualitative and semiquantitative changes were observed in the pyrograms of the samples collected during the accelerated ageing experiment. The Py-GC/MS chromatograms corresponding to the two shots are shown in Figure 4a,b for Gt0 and Gt1390h, to straightforwardly evidence the similarities and differences, while the corresponding peak identification is reported in Table 2.

The first Py-GC-MS shot of all the samples was expected to feature additives included in plastics formulations to improve or modify the chemical-physical and mechanical properties and performances of the polymer, that can be selectively detected with a thermal desorption analysis [34]. For instance, peak #9 (2,4-di-tert-butylphenol) is commonly used as a UV stabilizer and an antioxidant in plastics. The pyrogram at t0 and at 1390 h were both characterized by an unknown peak (#12) featuring ions already highlighted in the average mass spectrum collected for the region at lower temperatures in the EGA-MS thermogram (Figure S6a, Supplementary Materials). The specific species responsible for peak #12 could not be positively assigned since its mass spectrum was not present in any mass spectra library nor compatible with any of the antioxidants commonly used in ABS [45,46]. While the first shot pyrograms of Gt0, Gt8h, Gt24h, Gt36h, Gt48h, Gt120h and Gt240h did not present any other significant peaks, that of Gt1390h was widely different, evidencing very intense peaks ascribable to free monomers and oxidized species. The detection of styrene (#1) and α-methylstyrene (#4) in this desorption shot confirms the presence in the sample of a significant fraction of free monomer, resulting from the depolymerization of SAN, as suggested by both DSC and TGA analyses. Other products identified were all oxygen-containing aromatic species. Amongst them, benzaldehyde (#2), phenol (#3), benzoic acid (#4) and propoxybenzene (#6) are possibly related to the photo-degradation of the styrene units. Products #1–4 have indeed already been reported as thermal-oxidative degradation products of polystyrene released at 350 °C, detected by GC-MS and HPLC-DAD [36,47]. In addition, succinic anhydride (#5) and phthalic anhydride (#8) can result from oxidation undergone by butadiene units. These results were consistent with the FT-IR spectra (Figure S4, Supplementary Materials), in which all aged samples showed an increase in the intensity of the band at 1600–1800 cm^−1^ and at 3000–3400 cm^−1^, corresponding to the carbonyl and hydroxyl groups, respectively, derived from polymer oxidation. In particular, the FTIR spectrum of Gt1390h was characterized by a very intense carbonyl band at about 1740 cm^−1^ and another strong one at 1230 cm^−1^, presumably ascribable to the C-O-C band of ester and ether bonds and to the C-O bonds of aromatic alcohols, highlighting that the oxidation involves not only butadiene, but also the polystyrene fraction [48]. Succinic anhydride and phthalic anhydride, as evidenced by pyrolysis [49], may also contribute to the broad and intense carbonyl band.

The profile of the second shot was consistent with that of ABS reported in the literature [28] for all the samples. The pyrogram can be subdivided into three zones:

- The first one at lower retention times (2.8–10.7 min), dominated by the monomers of the constituting polymers (butadiene #14, acrylonitrile #15, styrene #1 and α-methylstyrene #2);
- The second zone in an intermediate time interval (11.6–16.2 min) characterized by a styrene dimer (3-butene-1,3-diyldibenzene (SS) #49), acrylonitrile dimer (pentanedinitrile, 2-methylene- (ANAN) #31) and hybrid acrylonitrile and styrene dimers (2-methylene-4-phenylbutanenitrile (ANS) #42, 4-phenylpent-4-enenitrile (SAN’) #44, 4-phenylpentanenitrile (SAN) #45);
- The third one at higher retention times (16.6–19.6 min) featured peaks related to styrene trimers (5-hexene-1,3,5-triyltribenzene (SSS) #58) and hybrid acrylonitrile and styrene trimers (2-methylene-4-phenethylpentanedinitrile (ANANS) #51, 2-methylene-4-phenylheptanedinitrile (ANSAN) #52, 2-(2-phenylallyl)pentanedinitrile (SANAN) #53, 2-methylene-4,6-diphenylhexanenitrile (ANSS) #53, 4,6-diphenylhept-6-enenitrile (SSAN) #55, 2-phenethyl-4-phenylpent-4-enenitrile (SASN) #56, 2-Phenethyl-4-phenylpent-4-enenitrile (SANS) #57).

**Figure 5:**
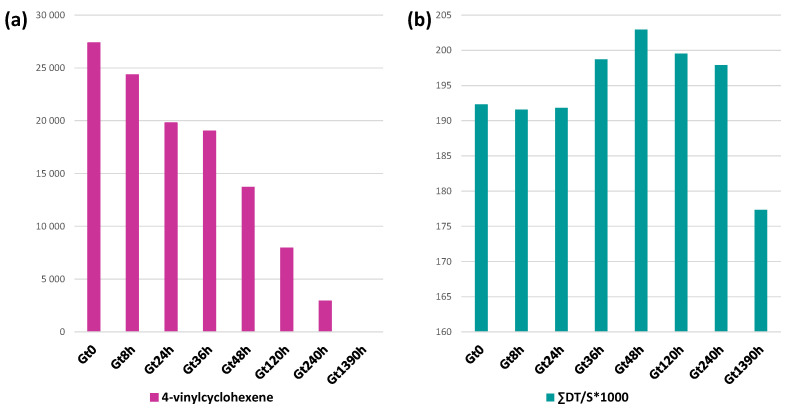
Histograms of the ratio of total ion chromatogram area in the Py-GC/MS chromatogram (shot 2) of (**a**) 4-vinylcyclohexene (normalized for sample weight) and (**b**) ratio between the sum of all styrene-containing dimers and trimers (ΣDT) over styrene (S) relative to the green brick at different ageing times (Gt0, Gt8h, Gt24h, Gt36h, Gt48h, Gt120h, Gt240h and Gt1390h).

The second shot pyrograms related to the aged samples mainly show semiquantitative changes, except for 4-vinylcyclohexene (#22) that was completely absent in the pyrogram of Gt1390h. 4-vinylcyclohexene is a butadiene dimer, formed as a pyrolysis product when the butadiene sequence is composed of at least two butadiene units directly connected, as in the case of the backbone of ABS [50,51]. The ageing leads to a decrease and then to the total disappearance of 4-vinylcyclohexene in Gt1390h (Figure 5a) as a consequence of the oxidation of PB, which prevents the formation of butadiene dimers. Moreover, some semiquantitative differences can be observed with ageing time, affecting the monomer to oligomer ratio. In particular, the area in the total ion chromatogram (TIC) of the three monomer peaks (AN, BU, S) were integrated for each sample. It is important to underline that the percentage values reported herein only refer to the relative peak areas and cannot account for the actual polymer composition in ABS (from the literature 40–60% styrene, 15–35% acrylonitrile and 5–30% butadiene [52]) due to the different pyrolysis yields and the instrumental response for each of them. Thus, we only discuss the relative intensity of signals, and not their absolute values. Keeping this caveat in mind, in the pyrolysis profile, the styrene peak was the most intense of the three (S 98.5%), with a 1.5% contribution to the summed areas from acrylonitrile (AN 1.0%) and a minor peak from butadiene (BU 0.5%). These percentage values did not significantly change in the ageing time, with S above 98% and BU and AN around 0.5–2%.

More interestingly, we observed a change in the ratio between the sum of all the styrene-containing oligomers (dimers SS, trimers SSS, hybrid acrylonitrile and styrene dimers (ANS, SAN′, SAN) and trimers (ANANS, ANSAN, SANAN, ANSS, SSAN, SANS)) and the monomer (ΣDT/S). This ratio remained constant in the first 24 h of ageing, slightly increased in the range 36–48 h, and then from 120 h decreased until it showed a sharp decline at 1390 h (Figure 5b). The initial slight increase may be connected to butadiene cross-linking, in agreement with the disappearance of the δCH trans butadiene band in the FT-IR spectrum between 24 and 36 h, leading to lower pyrolysis yields and thus to a lower amount of the S monomer amongst the products. After 120 h, the decrease in the ratio suggests the occurrence of SAN depolymerization, in agreement with the increase in the bands assigned to oxidized species in the FT-IR spectrum of Gt1390h, eventually resulting in an increase in plasticity as observed by DSC. The evidence that depolymerization was mainly affecting the SAN fraction of the polymer was highly consistent with the data obtained by TGA, DSC and EGA-MS.

## 4. Discussion

A combined approach based on thermal analytical and mass spectrometric techniques (TGA, DSC, EGA-MS, DS-Py-GC/MS), supported by external reflection FT-IR spectroscopy, was applied for the first time to investigate ABS photo-degradation in LEGO^®^ bricks; here, used as an example of industrially manufactured ABS objects. A rather prolonged photo-aging experiment (up to 1390 h), with respect to those reported in the literature, was performed to investigate phenomena not visible in the early stages of light exposure, which evidenced further thermal and compositional modifications in the polymer. The complementary information obtained by the thermoanalytical techniques was corroborated with external reflection FTIR spectroscopy assigning infrared bands related to on-going oxidation processes.

The ABS samples under analysis showed the typical composition reported in the literature [28], thoroughly characterized in terms of monomers, dimers and trimers by PY-GC/MS. Although no strict quantification can be performed for copolymers, the relative intensity of the pyrolysis peaks assigned to each monomer highlighted styrene as dominant, with a very minor amount of butadiene. The low percentage of the polybutadiene fraction was also confirmed by the TGA and DSC curves collected, which respectively featured a single thermal degradation step (Tmax = 424 °C) and a single glass transition (Tg = 104 °C) ascribable to SAN, while the presence of the PB portion could only be roughly inferred by the small residue at 600 °C (1.1%) to which the TiO_2_ present in the samples also contributed.

As regards ABS photo-ageing: DSC evidenced a shift towards lower temperatures in the Tg (ΔT = 19 °C) of the sample; TGA evidenced an increase in the % residue at 600 °C and, and, together with EGA, a decrease in the maximum temperature of the mass loss at a higher irradiation time; the semiquantitative analysis of the pyrolysis results indicated a relevant decrease in the ratio between the sum of all styrene-containing dimers and trimers over styrene (ΣDT/S). All these results are consistent with an increase in the plasticity of the material due to a depolymerization process mainly affecting the SAN portion. The decreasing trend with ageing, up to the total disappearance of 4-vinylcyclohexene, a butadiene dimer, in the pyrolysis profiles, supported PB crosslinking, consistent with the increase in the residue in TGA and with the disappearance of the bands due to δCH trans of butadiene and to C=C aliphatic of vinyl-butadiene in the FT-IR spectra. Moreover, the thermal-oxidative ageing products related to PS (styrene, benzaldehyde, phenol, benzoic acid, α-methylstyrene, propoxybenzene) and PB (succinic anhydride and phthalic anhydride), some of which have never before been documented, were detected by DS-PY-GC/MS performed at 350 °C (desorption step) and supported by the detection of strong bands due to carbonyl and hydroxyl groups as well as those relative to the C-O-C band of ester bonds and that of the C-O of aromatic alcohols in the FT-IR spectra, pointing toward a further oxidation process occurring.

**Figure 6:**
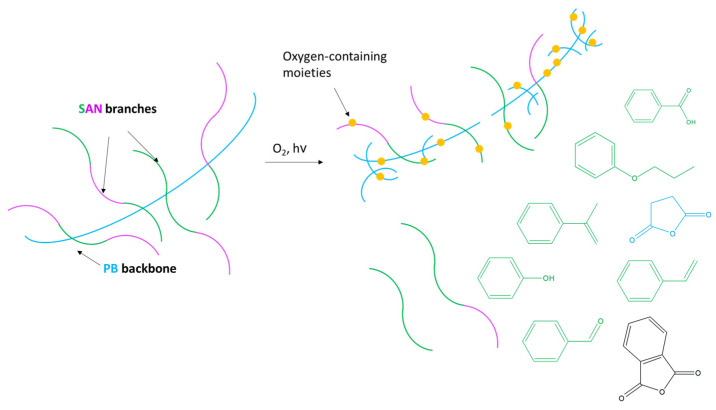
Scheme of the hypothesized process for ABS photo-oxidation.

Considering the results collected with the presented analytical approach, the following photo-degradation pathway induced by accelerated ageing was suggested and sketched in Figure 6. At the first stage of ageing, the process affects the superficial layers of the sample in contact with light and oxygen. PB photo-oxidation leads to the formation of free radicals and, after reaction with oxygen, of oxo- and peroxyradicals [53,54]. These products, generally highly unstable, may convert to more stable secondary products and rearrangements that can be identified as succinic anhydride and phthalic anhydride pyrolysis products. The onset of such oxidation processes was successfully monitored by external reflection FT-IR and by the decrease in the 4-vinylcyclohexene peak in Py-GC/MS, never evaluated before for the study of ABS photo-oxidation. In conditions of prolonged light exposure (above 120 h), photo-aging induced further chain scission reactions leading to the depolymerization of SAN [43,55]. This phenomenon has rarely been observed, possibly due to the relatively long photo-ageing time required to induce SAN depolymerization in ABS.

## 5. Conclusions

In this study, our novel comprehensive investigation protocol was able to provide detailed and complementary information on ABS photo-degradation in artworks. Specifically, a two-step pathway was proposed for ABS photo-degradation. Spectral features highlighted by external reflection FT-IR spectroscopy and the relative peak area of 4-vinylcyclohexene, a pyrolysis product never mentioned in ABS studies, were used to monitor the oxidation of the polybutadiene fraction (PB). In a second step, through DSC and the evaluation of the oligomer to monomer ratios in the pyrograms, we monitored the depolymerization of the styrene-acrylonitrile (SAN) fraction.

Despite the selected analytical approach being mainly based on bulk thermoanalytical techniques, generally only able to detect relevant changes at higher irradiation times, we were able to monitor the onset of the changes in the polymer structure, as corroborated by FTIR. In this regard, the choice of working on powdered samples, providing a high surface to volume ratio, was successful and opens new perspectives to speed up the photo-aging of samples for the study of compositional changes occurring at the surface. In this way, the issue of non-representative sampling will be solved and the study of synthetic materials in design objects may be performed in reasonable time frames by thermo-analytical techniques.

This study constituted a pilot study to be expanded to further classes of polymers encountered in heritage objects and museum collections.

## Figures and Tables

**Table 1:** Glass transition temperature from DSC (Tg), maximum temperature of the degradation step from TGA (Tmax); peak temperature of the total current thermogram profile from EGA/MS (Tp), for the samples from the green brick after different ageing times (Gt0, Gt8h, Gt24h, Gt36h, Gt48h, Gt120h, Gt240h and Gt1390h).

	DSC	TGA	EGA/MS
	Tg (°C)	Tmax (°C) Weight Loss (°C/% *w*/*w*)	Tp (°C)
**Gt0**	104	424	431
		99%	
**Gt8h**	104	422	431
		99%	
**Gt24h**	98	422	430
		100%	
**Gt36h**	98	422	430
		100%	
**Gt48h**	94	422	429
		98%	
**Gt120h**	94	420	429
		97%	
**Gt240h**	93	419	423
		97%	
**Gt1390h**	85	403	413
		92%	

**Table 2:** Main pyrolysis products detected in the first and second shot Py-GC-MS of Gt0 and Gt1390h (Figure 4a,b).

	#	tr (Min)	Compound
First shot	1	9.5	Styrene
	2	10.5	Benzaldehyde
	3	10.7	Phenol
	4	10.7	α-Methylstyrene
	5	11.0	Succinic anhydride
	6	11.5	Benzene, propoxy
	7	12.4	Benzoic acid
	8	13.7	Phthalic anhydride
	9	14.9	2,4-Di-tert-butylphenol
	10	17.4	2-[1-(4-Cyano-1,2,3,4-tetrahydronaphthyl)]propanenitrile
	11	17.5	3-[1-(4-Cyano-1,2,3,4-tetrahydronaphthyl)]propanenitrile
	12	18.9	*m*/*z* = 77, 91, 105, 115, 129, 207
Second shot	13	2.3	Carbon dioxide
	14	2.5	1,3-Butadiene
	15	2.8	Acrylonitrile
	16	3.5	Methylacrylonitrile
	17	4.2	Benzene
	18	5.0	2-Pentenenitrile
	19	7.0	Toluene
	20	7.6	4-Heptenal, (Z)-
	21	8.3	2-Cyclopenten-1-one
	22	8.5	4-vinylcyclohexene
	23	8.9	Ethylbenzene
	24	9.2	Cyclohexen-1-carbonitrile
	1	9.4	Styrene
	25	10	Benzene, (1-methylethyl)-
	26	10.3	Benzene, 2-propenyl-
	27	10.4	Benzene, propyl-
	2	10.5	Benzaldehyde
	4	10.7	α-Methylstyrene
	28	11.3	Benzene, 3-butenyl-
	29	11.4	Benzene, 1-ethynyl-4-methyl-
	30	11.5	Benzene, (1-methylenepropyl)-
	31	11.6	Pentanedinitrile, 2-methylene- (ANAN)
	32	11.7	Acetophenone
	33	12.3	Benzyl nitrile
	34	12.3	Benzofuran, 2,3-dihydro-
	35	12.4	Naphthalene, 1,2-dihydro-
	36	12.5	2-Phenylpropenal
	37	12.6	2-Propenenitrile, 3-phenyl-, (E)-
	38	12.7	Naphthalene
	39	12.8	4-Vinylphenol
	40	13.1	Benzenepropanenitrile
	41	13.2	Benzenepropanenitrile, α-methylene-
	42	13.7	2-methylene-4-phenylbutanenitrile (ANS)
	43	14.0	Benzene, (1-methyl-3-butenyl)-
	44	14.3	4-Phenylpent-4-enenitrile (SAN’)
	45	14.4	4-Phenylpentanenitrile (SAN)
	46	15	Bibenzyl
	47	15.2	Benzene, 1,1′-(1-methyl-1,2-ethanediyl)bis-
	48	15.8	Benzene, 1,1′-(1,3-propanediyl)bis-
	49	16.2	3-Butene-1,3-diyldibenzene (SS)
	50	16.3	1,2-Diphenylcyclopropane
	51	16.6	2-Methylene-4-phenethylpentanedinitrile (ANANS)
	52	16.8	2-Methylene-4-phenylheptanedinitrile (ANSAN)
	53	16.9	2-(2-Phenylallyl)pentanedinitrile (SANAN)
	54	17.3	Hex-1-ene,2,5-diphenyl-
	55	18.2	2-Methylene-4,6-diphenylhexanenitrile (ANSS)
	56	18.4	4,6-Diphenylhept-6-enenitrile (SSAN)
	57	18.5	2-Phenethyl-4-phenylpent-4-enenitrile (SANS)
	58	19.6	5-Hexene-1,3,5-triyltribenzene (SSS)

## Data Availability

The data that support the findings of this study are available from the corresponding author, I.D., upon reasonable request.

## Supplementary Materials

The following supporting information can be downloaded at: https://www.mdpi.com/article/10.3390/polym15153267/s1, Figure S1: Poly(acrylonitrile butadiene styrene): molecular structure of monomers and schematic representation of polymer structure; Figure S2: (a) Raman spectra of unaged green (Gt0) and white samples (Wt0) collected with 785 nm laser. The bands relative to PG7 and PG26 are labelled with green arrows; those due to TiO_2_ with grey arrows; while those ascribable to ABS with asterisks; Figure S3: (a) Thermogravimetric curve acquired under nitrogen flow at 10 °C/min heating rate for the unaged white sample (Wt0) and (b) corresponding derivative; Figure S4: FT-IR spectra recorded in external reflection mode for green samples at different ageing times (Gt0, Gt8h, Gt24h, Gt36h, Gt48h, Gt120h, Gt240h and Gt1390h). All spectra are presented in the same scale and are stacked for purposes of clarity; Figure S5: DSC curve of the heat flow normalized in the second heating vs. temperature for the unaged white sample (Wt0) compared with unaged green sample (Gt0); Figure S6: DSC curves of the heat flow normalized in the second heating vs. temperature for PG7 and PG36; Figure S7: Average mass spectra obtained from the total ion thermograms of Gt0 sample in (a) the secondary thermal degradation zone (14.5–20.7 min, 200–260 °C), (b) main degradation thermal zone (31.3–46.1 min, 360–510 °C). The characteristic ions of styrene are highlighted in green, those related to acrylonitrile-styrene trimers in brown and the ions in common to both components in bold.

## References

[1] Chiantore O., Lazzari M. (2001). Photo-oxidative stability of Paraloid acrylic protective polymers. Polymer.

[2] Cardiano P., Sergi S., Lazzari M., Piraino P. (2002). Epoxy–silica polymers as restoration materials. Polymer.

[3] Learner T. (2004). Analysis of Modern Paints.

[4] Shashoua Y. (2009). Conservation of Plastics—Materials Science, Degradation and Preservation.

[5] Van Oosten T.B. (2022). Properties of Plastics: A Guide for Conservators.

[6] Lazzari M., Reggio D. (2021). What Fate for Plastics in Artworks? An Overview of Their Identification and Degradative Behaviour. Polymers.

[7] Rosi F., Miliani C., Gardner P., Chieli A., Romani A., Ciabatta M., Trevisan R., Ferriani B., Richardson E., Cartechini L. (2021). Unveiling the composition of historical plastics through non-invasive reflection FT-IR spectroscopy in the extended near- and mid-Infrared spectral range. Anal. Chim. Acta.

[8] E-RIHS.it (n.d.). www.e-rihs.it.

[9] Alfredo Campo E. (2008). Selection of Polymeric Materials: How to Select Design Properties from Different Standards.

[10] Rutkowski J.V., Levin B.C. (1986). Acrylonitrile-butadiene-styrene copolymers (ABS): Pyrolysis and combustion products and their toxicity—A review of the literature. Fire Mater..

[11] Wyzgoski M.G. (1976). Effects of oven aging on ABS, poly(acrylonitrile-butadiene-styrene). Polym. Eng. Sci..

[12] Streibel T., Geißler R., Saraji-Bozorgzad M., Sklorz M., Kaisersberger E., Denner T., Zimmermann R. (2009). Evolved gas analysis (EGA) in TG and DSC with single photon ionisation mass spectrometry (SPI-MS): Molecular organic signatures from pyrolysis of soft and hard wood, coal, crude oil and ABS polymer. J. Therm. Anal. Calorim..

[13] Polli H., Pontes L.A.M., Araujo A.S., Barros J.M.F., Fernandes V.J. (2009). Degradation behavior and kinetic study of ABS polymer. J. Therm. Anal. Calorim..

[14] Shapi M. (1991). TG and DSC studies of some thermal properties and stability aspects of poly(acrylonitrile butadiene styrene), polystyrene and poly(acrylonitrile styrene) plastics. Thermochim. Acta.

[15] Yang M.-H. (2000). The thermal degradation of acrylonitrile-butadiene-styrene terpolymer under various gas conditions. Polym. Test..

[16] di Cortemiglia M., Camino G., Costa L., Guaita M. (1985). Thermal degradation of ABS. Thermochim. Acta.

[17] Fǎtu D., Geambaş G., Segal E., Budrugeac P., Ciutacu S. (1989). On the thermal decomposition of the copolymer ABS and of nylon polyamide. Thermochim. Acta.

[18] Wu X., Bourbigot S., Li K., Zou Y. (2022). Co-pyrolysis characteristics and flammability of polylactic acid and acrylonitrile-butadiene-styrene plastic blend using TG, temperature-dependent FTIR, Py-GC/MS and cone calorimeter analyses. Fire Saf. J..

[19] Suzuki M., Wilkie C.A. (1995). The thermal degradation of acrylonitrile-butadiene-styrene terploymer grafted with methacrylic acid. Polym. Degrad. Stab..

[20] Suzuki M., Costa I.L., Pereira P.H., Claro A.M., Amaral NC D., Barud HD S., Ribeiro R.B., Mulinari D.R. (2023). 3D-printing pen from valorization of pine cone residues as reinforcement in acrylonitrile butadiene styrene (ABS): Microstructure and thermal properties. J. Thermoplas. Compos. Mater..

[21] Du A.-K., Zhou Q., van Kasteren J.M., Wang Y.-Z. (2011). Fuel oil from ABS using a tandem PEG-enhanced denitrogenation–pyrolysis method: Thermal degradation of denitrogenated ABS. J. Anal. Appl. Pyrolysis.

[22] Liu G., Liao Y., Ma X. (2017). Thermal behavior of vehicle plastic blends contained acrylonitrile-butadiene-styrene (ABS) in pyrolysis using TG-FTIR. Waste Manag..

[23] Herrera M., Matuschek G., Kettrup A. (2003). Fast identification of polymer additives by pyrolysis-gas chromatography/mass spectrometry. J. Anal. Appl. Pyrolysis.

[24] Blom H., Yeh R., Wojnarowski R., Ling M. (2006). Detection of degradation of ABS materials via DSC. J. Therm. Anal. Calorim..

[25] Belhaneche-Bensemra N., Bedda A., Belaabed B. (2003). Study of the properties of rigid and plasticized PVC/PMMA blends. Macromolecular Symposia.

[26] Duh Y.-S., Ho T.-C., Chen J.-R., Kao C.-S. (2010). Study on Exothermic Oxidation of Acrylonitrile-butadiene-styrene (ABS) Resin Powder with Application to ABS Processing Safety. Polymers.

[27] Bair H.E., Boyle D.J., Kelleher P.G. (1980). The effects of light and heat on the rubber content and impact strength of acrylonitrile-butadiene-styrene. Polym. Eng. Sci..

[28] Tsuge S., Ohtani H., Watanabe C. (2011). Pyrolysis—GC/MS Data Book of Synthetic Polymers.

[29] La Nasa J., Biale G., Mattonai M., Modugno F. (2021). Microwave-assisted solvent extraction and double-shot analytical pyrolysis for the quali-quantitation of plasticizers and microplastics in beach sand samples. J. Hazard. Mater..

[30] La Nasa J., Biale G., Ferriani B., Colombini M.P., Modugno F. (2018). A pyrolysis approach for characterizing and assessing degradation of polyurethane foam in cultural heritage objects. J. Anal. Appl. Pyrolysis.

[31] La Nasa J., Biale G., Sabatini F., Degano I., Colombini M.P., Modugno F. (2019). Synthetic materials in art: A new comprehensive approach for the characterization of multi-material artworks by analytical pyrolysis. Herit. Sci..

[32] Sabatini F., Manariti A., di Girolamo F., Bonaduce I., Tozzi L., Rava A., Colombini M.P., Lluveras-Tenorio A. (2020). Painting on polyurethane foam: “Composizione-Superficie Lunare” by Giulio Turcato. Microchem. J..

[33] Micheluz A., Angelin E.M., Sawitzki J., Pamplona M. (2022). Plastics in robots: A degradation study of a humanoid skin mask made of soft urethane elastomer. Herit. Sci..

[34] Pintus V., Viana C., Angelin E.M., De Sá S.F., Wienland K., Sterflinger K., Ferreira J.L. (2022). Applicability of single-shot and double-shot Py-GC/MS for the detection of components in vinyl acetate-based emulsions used in modern-contemporary art. J. Anal. Appl. Pyrolysis.

[35] Akoueson F., Chbib C., Monchy S., Paul-Pont I., Doyen P., Dehaut A., Duflos G. (2021). Identification and quantification of plastic additives using pyrolysis-GC/MS: A review. Sci. Total. Environ..

[36] Levin B.C. (1987). A summary of the NBS literature reviews on the chemical nature and toxicity of the pyrolysis and combustion products from seven plastics: Acrylonitrile-butadiene-styrenes (ABS), nylons, polyesters, polyethylenes, polystyrenes, poly(vinyl chlorides) and rigid polyurethane foams. Fire Mater..

[37] Day M., Cooney J., Touchette-Barrette C., Sheehan S. (1999). Pyrolysis of mixed plastics used in the electronics industry. J. Anal. Appl. Pyrolysis.

[38] Szabo E., Olah M., Ronkay F., Miskolczi N., Blazso M. (2011). Characterization of the liquid product recovered through pyrolysis of PMMA–ABS waste. J. Anal. Appl. Pyrolysis.

[39] Romanova N., Shafigullin L., Gabdrakhmanov A., Buyatova S. (2019). Thermal properties of products based on ABS/PC. MATEC Web Conf..

[40] Andersen E., Bertelsen L.H., Salomonsen M., Kristensen M., Kybelund P., Sørensen M.B., Hinge M. (2020). Accelerating effect of pigments on poly(acrylonitrile butadiene styrene) degradation. Polym. Degrad. Stab..

[41] Yang S., Castilleja J.R., Barrera E., Lozano K. (2004). Thermal analysis of an acrylonitrile–butadiene–styrene/SWNT composite. Polym. Degrad. Stab..

[42] Duce C., Bernazzani L., Bramanti E., Spepi A., Colombini M., Tiné M. (2014). Alkyd artists’ paints: Do pigments affect the stability of the resin? A TG and DSC study on fast-drying oil colours. Polym. Degrad. Stab..

[43] Suzuki M., Wilkie C.A. (1995). The thermal degradation of acrylonitrile-butadiene-styrene terpolymer as studied by TGA/FTIR. Polym. Degrad. Stab..

[44] Martel B. (1988). Charring processes in thermoplastic polymers: Effect of condensed phase oxidation on the formation of chars in pure polymers. J. Appl. Polym. Sci..

[45] Wang Y., Chen M., Lan M., Li Z., Lu S., Wu G. (2020). GM-Improved Antiaging Effect of Acrylonitrile Butadiene Styrene in Different Thermal Environments. Polymers.

[46] Zhu Z.F., Wen J., Zhang Q., Yuan S., Yin L. (2020). The effect of antioxidant 1010 on the thermo-oxidative aging properties of polycarbonate/acrylonitrile–butadiene–styrene blends. J. Phys. Conf. Ser..

[47] Piton M., Rivaton A. (1997). Photo-oxidation of ABS at long wavelengths (λ > 300 nm). Polym. Degrad. Stab..

[48] Santos R.M., Botelho G.L., Machado A.V. (2010). Artificial and natural weathering of ABS. J. Appl. Polym. Sci..

[49] Hase Y., Davanzo C.U., Kawai K., Sala O. (1976). The vibrational spectra of phthalic anhydride. J. Mol. Struct..

[50] Choi S.-S., Han D.-H. (2007). Pyrolysis behaviors of poly(acrylonitrile-co-butadiene) with differing microstructures. J. Anal. Appl. Pyrolysis.

[51] McNeill I., Stevenson W. (1985). The effect of 4-vinylcyclohexene on the degradation of polystyrene and on the polymerisation of styrene. Polym. Degrad. Stab..

[52] Shi Y., Yan C., Zhou Y., Wu J., Wang Y., Yu S., Chen Y., Shi Y., Yan C., Zhou Y., Wu J., Wang Y., Yu S., Chen Y. (2021). Polymer materials for additive manufacturing—Powder materials. Materials for Additive Manufacturing.

[53] Saviello D., Andena L., Gastaldi D., Toniolo L., Goidanich S. (2018). Multi-analytical approach for the morphological, molecular, and mechanical characterization after photo-oxidation of polymers used in artworks. J. Appl. Polym. Sci..

[54] Tiganis B., Burn L., Davis P., Hill A. (2002). Thermal degradation of acrylonitrile–butadiene–styrene (ABS) blends. Polym. Degrad. Stab..

[55] Jang B.N., Wilkie C.A. (2005). The effects of clay on the thermal degradation behavior of poly(styrene-co-acrylonitirile). Polymer.

