# Supplementary material for: A Thermal Analytical Study of LEGO® Bricks for Investigating Light-Stability of ABS
Source: Polymers (Basel). 2023 Jul 31;15(15):3267. doi: 10.3390/polym15153267 (PMC10422395; doi:10.3390/polym15153267)
Supplement: Supplementary file 1 [file polymers-15-03267-s001.zip › polymers-2489024-supplementary.pdf]

Supplementary materials for

A thermal analytical study of LEGO® bricks for investigating light-stability of ABS

Francesca Sabatini [1#], Silvia Pizzimenti [2], Irene Bargagli [1], Ilaria Degano [2,*], Celia Duce [2], Laura Cartechini [1], Francesca Modugno [2,*] and Francesca Rosi [1]

[1] 1 Institute of Chemical Science and Technologies "G. Natta" (CNR-SCITEC), Via Elce di Sotto 8, 01628, Perugia, (Italy); f.sabatini4@gmail.com; irene.bargagli@studenti.unipg.it; laura.cartechini@cnr.it; francesca.rosi@cnr.it

[2] Department of Chemistry and Industrial Chemistry, University of Pisa, Via G. Moruzzi 13, 56124, Pisa, (Italy); silvia.pizzimenti@gmail.com; ilaria.degano@unipi.it; francesca.modugno@unipi.it

# Current affiliation: Department of Earth and Environmental Sciences, University of Milano-Bicocca, Piazza della Scienza 1, 20126 Milan (Italy); f.sabatini4@gmail.com

* Correspondence: ilaria.degano@unipi.it; francesca.modugno@unipi.it; Tel.: +390502219255 (ID)

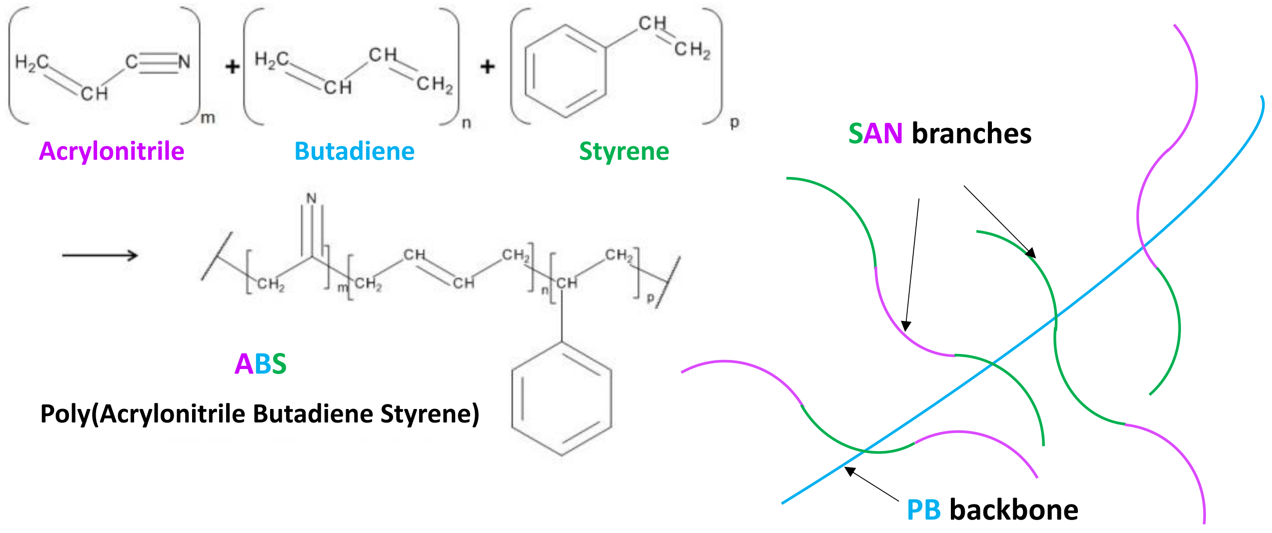

Figure S1: Poly(Acrylonitrile Butadiene Styrene): molecular structure of monomers and schematic representation of polymer structure.

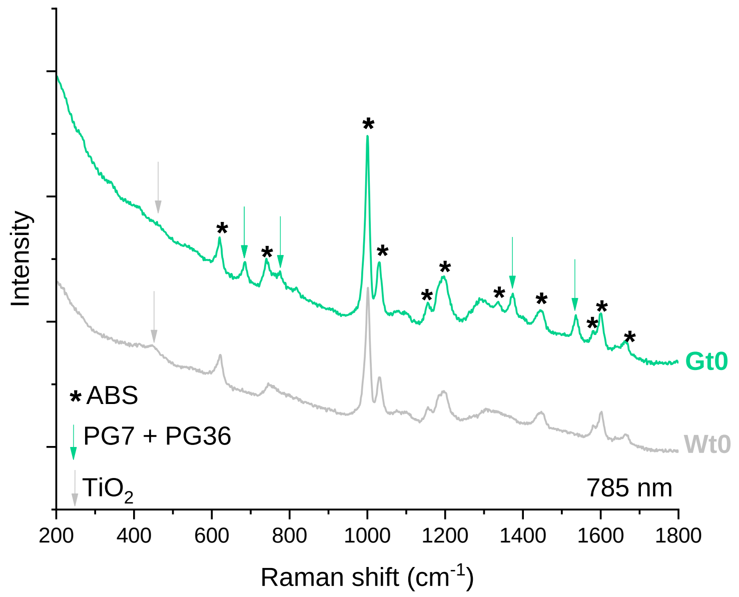

Figure S2: a) Raman spectra of unaged green (Gt0) and white sample (Wt0) collected with 785 nm laser. The bands relative to PG7 and PG26 are labelled with green arrows, those due to TiO_2 with grey arrows while those ascribable to ABS with asterisks.

Raman apparatus:

A JASCO NRS-3100 Benchtop Spectrophotometer was used. The spectra were acquired using an optical microscope with x100 lens, a CCD (Charge Coupled Devise) detector cooled to -53 °C by Peltier system, and an excitation wavelength at 785 nm emitted by a laser diode using a grating of 600 l/mm.

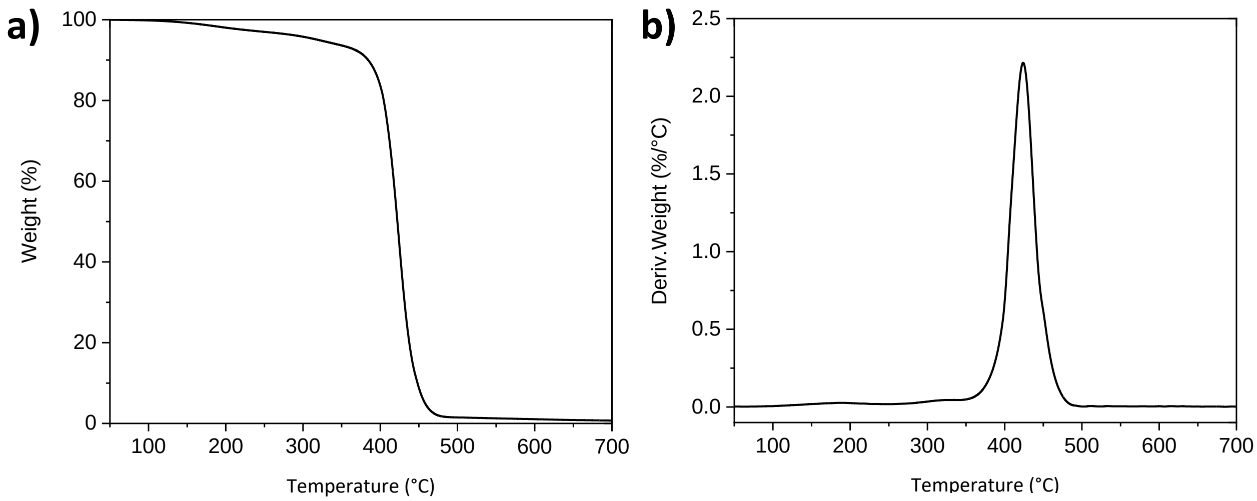

Figure S3: a) Thermogravimetric curve acquired under nitrogen flow at 10 °C/min heating rate for the unaged white sample (Wt0) and **b)** corresponding derivative.

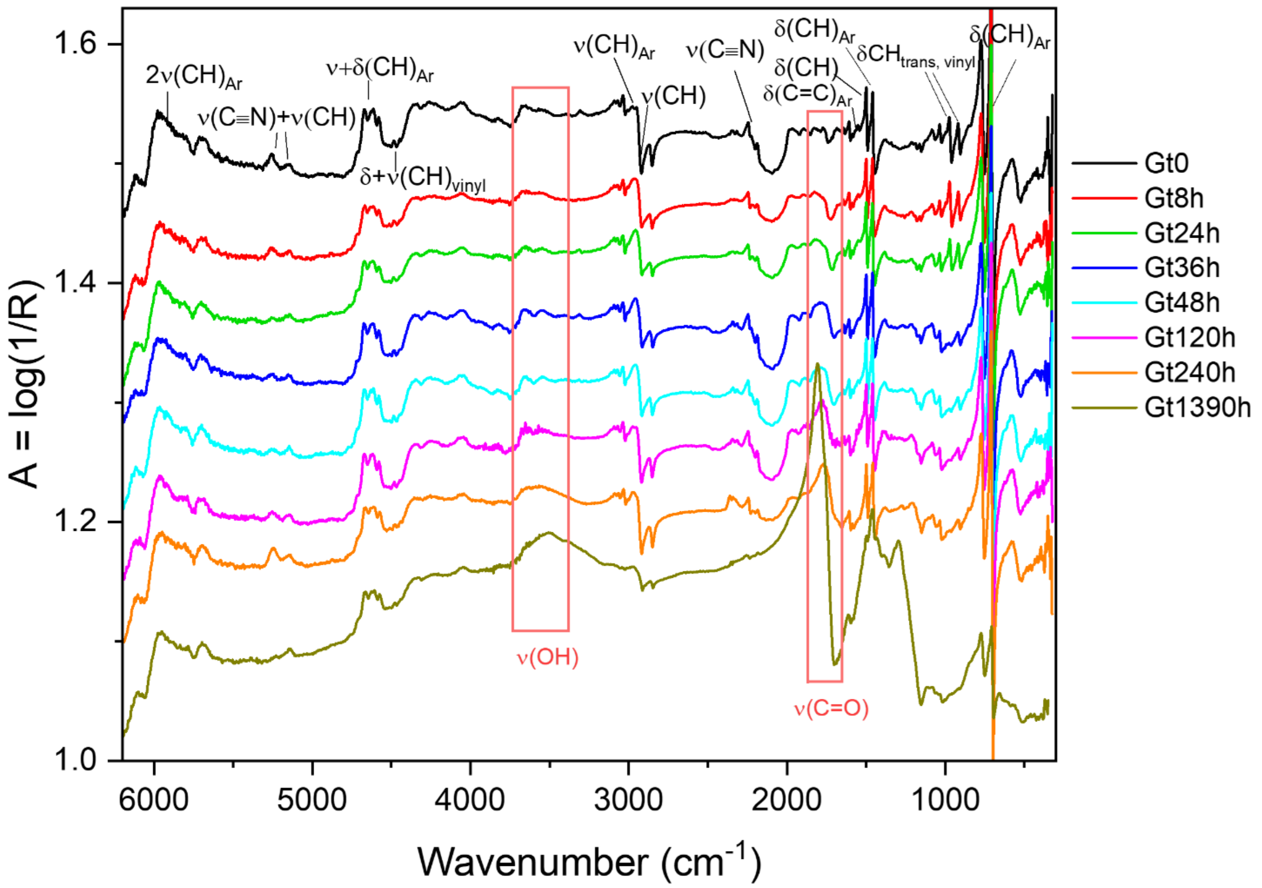

Figure S4: FT-IR spectra recorded in external reflection mode for green samples at different ageing times (Gt0, Gt8h, Gt24h, Gt36h, Gt48h, Gt120h, Gt240h and Gt1390h). All spectra are presented in the same scale and are stacked for purposes of clarity.

External reflection FT-IR apparatus:

External reflection FT-IR was performed with a portable FT-IR spectrometer (ALPHA, Bruker Optics, Germany/USA—MA) equipped with a SiC globar source, a “rock solid”-design interferometer (with gold mirrors) and a DLaTGS detector. The external reflectance module has an optical layout of 22°/22°. Pseudo-absorption spectra [log(1/R); R = reflectance] were acquired from areas of about 20 mm^2, in the spectral range of 7000–375 cm^{-1}, at a spectral resolution of 4 cm^{-1} and using 144 scans. Spectra from a gold flat mirror were used as background. Three spectra were acquired on different areas of the sample. All data were collected using Opus software (v7.2).

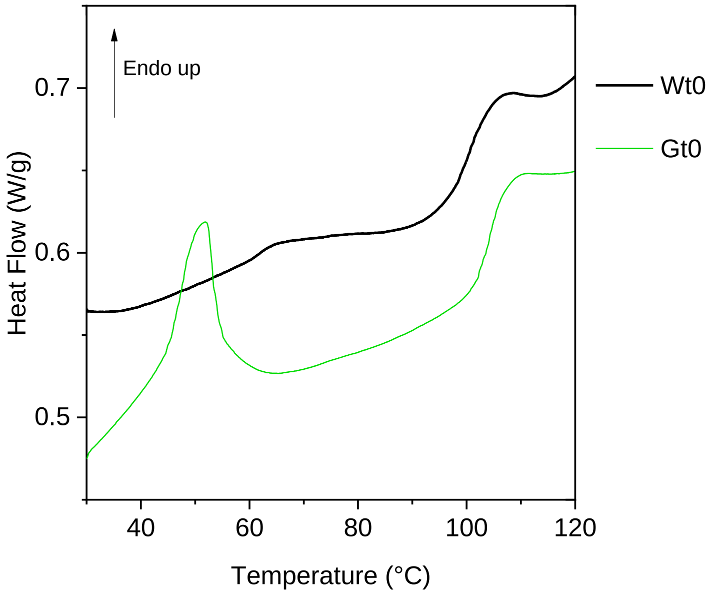

Figure S5: DSC curve of the heat flow normalized in the second heating vs. temperature for the unaged white sample (Wt0) compared with unaged green sample (Gt0).

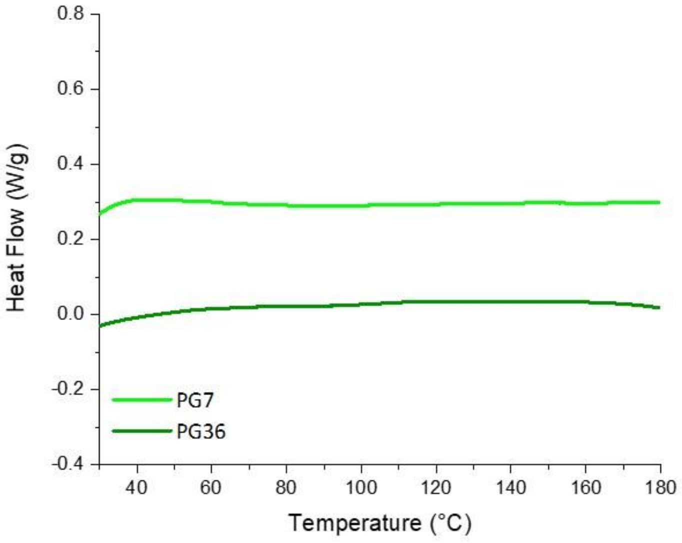

Figure S6: DSC curves of the heat flow normalized in the second heating vs. temperature for PG7 and PG36.

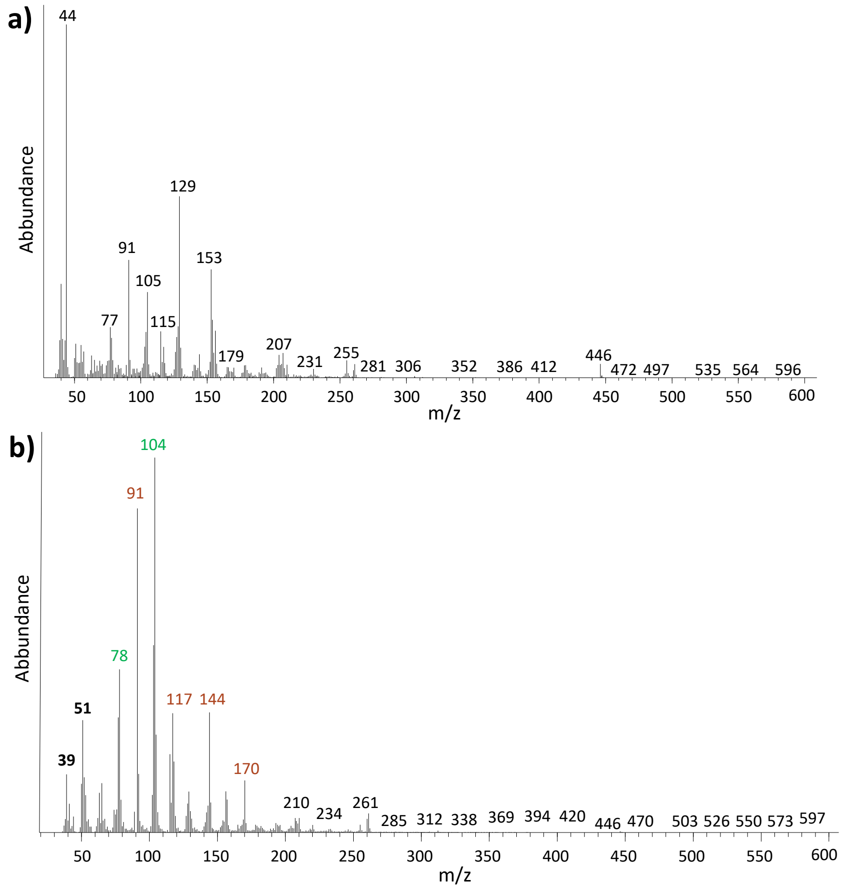

Figure S7: Average mass spectra obtained from the Total Ion Thermograms of Gt0 sample in **a)** the secondary thermal degradation zone (14.5-20.7 min, 200-260 °C); **b)** the main degradation thermal zone (31.3-46.1 min, 360-510 °C). The characteristic ions of styrene are highlighted in green, those related to acrylonitrile-styrene trimers in brown and the ions in common to both components in bold.
